# Giant lipoma in the hand: A case report

**DOI:** 10.1097/MD.0000000000018434

**Published:** 2019-12-27

**Authors:** Kwang Seog Kim, Hyeok Lee, Dong Seob Lim, Jae Ha Hwang, Sam Yong Lee

**Affiliations:** Department of Plastic and Reconstructive Surgery, Chonnam National University Medical School, Gwangju, Republic of Korea.

**Keywords:** hand, lipoma, microsurgery

## Abstract

**Rationale:**

Although lipomas are the most common benign form of soft tissue tumor in the body, giant lipomas of the hand, defined as >5 cm in diameter, are extremely rare.

**Patient concerns:**

A 49-year-old man presented with a soft and fixed lump in the left hypothenar area. The mass was not tender, but it was associated with symptoms of tingling sensation and paresthesia in the left ring and little fingers that had lasted for 4 years.

**Diagnoses:**

Preoperative image studies revealed an encapsulated and multilobulated mass, which measured 8 cm × 5 cm × 2 cm. Basic histologic examination identified the specimen as a lipoma and further immunohistochemical studies ruled out the possibility of malignancy.

**Interventions:**

To enable a complete excision of the mass, the palmar digital branch of the ulnar nerve for the little finger passing through the mass was temporarily transected. After complete excision of the mass, the branch was coapted again under microscopy.

**Outcomes:**

Complete sensory recovery was achieved 6 months after surgery, without any sign of recurrence.

**Lessons:**

Although giant lipomas in the hand can extend to vital components such as neurovascular structures, muscles, and tendons, meticulous en bloc resection can provide excellent results without any complications.

## 1 Introduction

Lipomas are the most common benign form of soft tissue tumor in the body.^[1]^ Although they are commonly found on the upper extremity, their occurrence in the hand is rare.^[2]^ Giant lipomas of the hand, defined as >5 cm in diameter, are extremely rare.[^3^,^4^] In this report, the authors present a patient with a giant lipoma on the palmar side of a hand.

## 2 Case presentation

**Figure 1:**
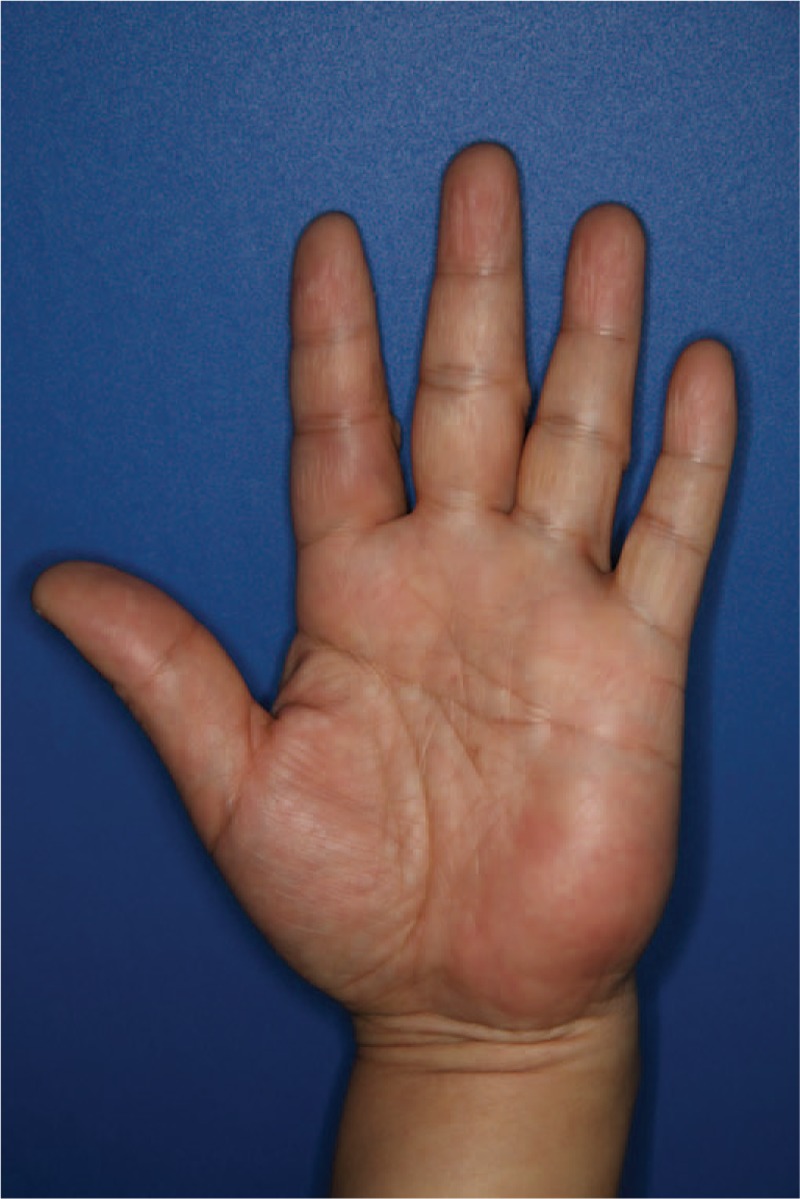
Preoperative photograph of the lipoma at the hypothenar area.

**Figure 2:**
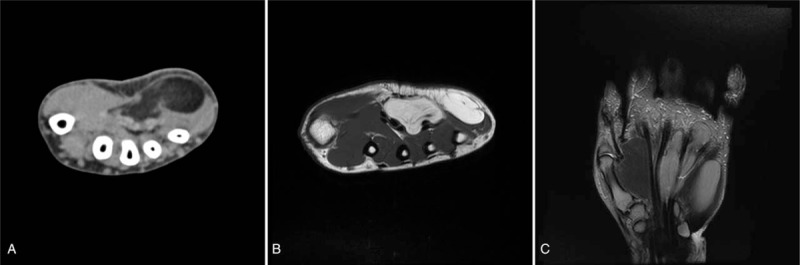
Preoperative computed tomography (A) and magnetic resonance imaging (B, C) scans of the mass. Preoperative image studies revealed an encapsulated and multilobulated mass compatible with a lipoma, which measured 8 cm × 5 cm × 2 cm.

A 49-year-old man presented with a soft and fixed lump in the left hypothenar area (Fig. 1). The mass was not tender, but it was associated with symptoms of tingling sensation and paresthesia in the left ring and little fingers that had lasted for 4 years. Preoperative image studies revealed an encapsulated and multilobulated mass compatible with a lipoma, which measured 8 cm × 5 cm × 2 cm (Fig. 2).

**Figure 3:**
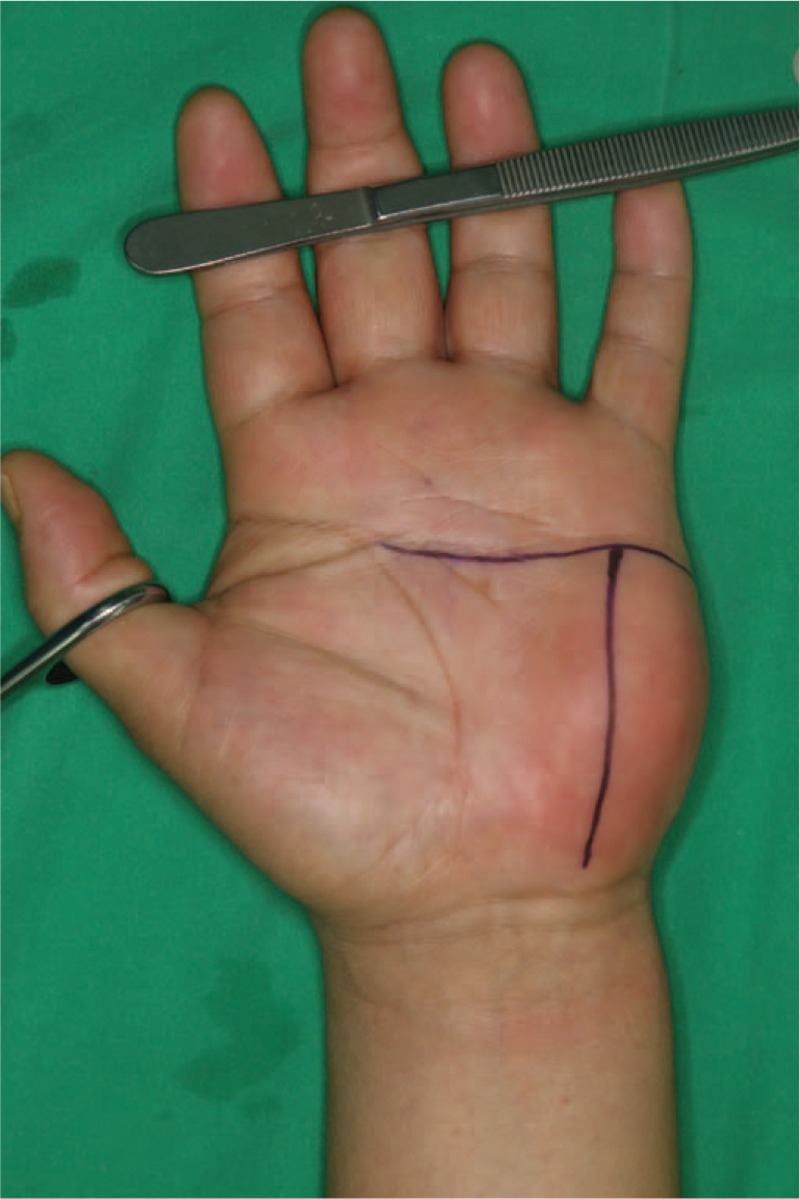
Incision line. The mass was operated by a T-shaped skin incision.

**Figure 4:**
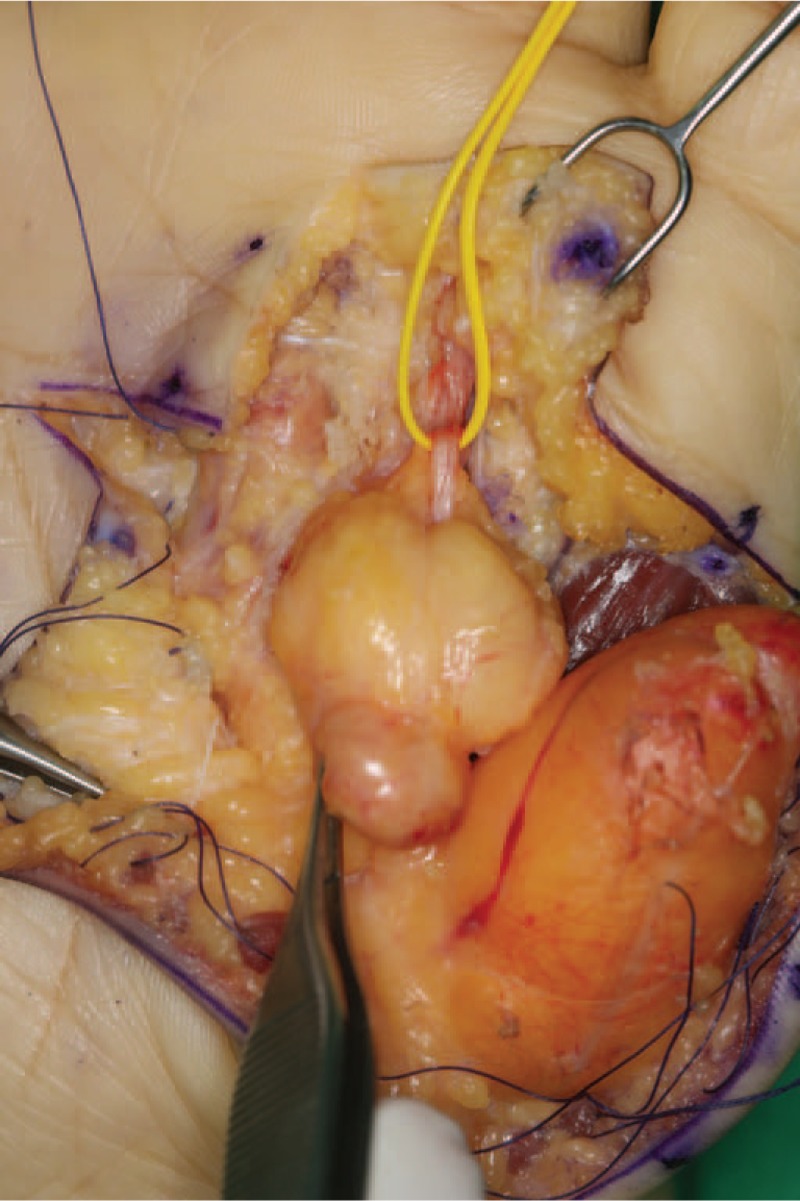
Intraoperative photograph. The palmar digital branches of the ulnar nerve for the ring and little fingers passed through the mass and the branch for the little finger was firmly attached to it.

**Figure 5:**
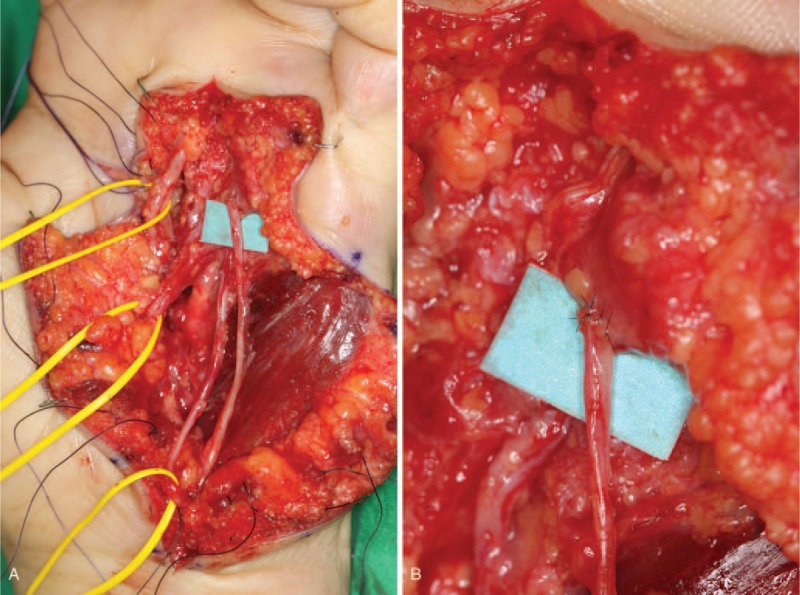
Intraoperative photographs just after complete excision of the mass. The palmar digital branch of the ulnar nerve for the little finger was temporarily transected and coapted again under microscopy.

Under general anesthesia, the mass was operated by a T-shaped skin incision (Fig. 3). The mass was mainly located in the subcutaneous layer, however, deep extensions were seen reaching into the carpal tunnel, the hypothenar muscles, and intertendinous spaces between the left index and little fingers. The palmar digital branches of the ulnar nerve for the ring and little fingers passed through the mass and the branch for the little finger was firmly attached to it (Fig. 4). To enable a complete excision of the mass, the branch for the little finger was temporarily transected. After complete excision of the mass, the transected branch was coapted again under microscopy (Fig. 5).

**Figure 6:**
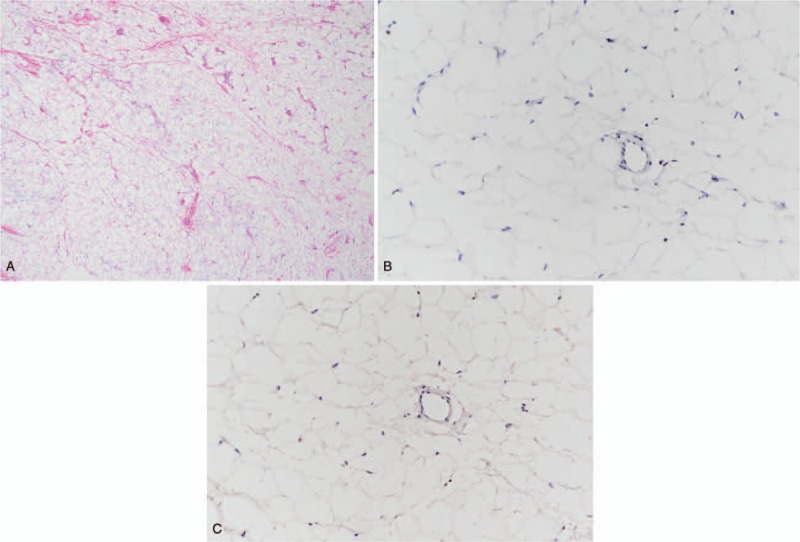
Histopathological images of the mass (A: H&E 40×, B: CDK4 200×, C: MDM2 200×). Histopathological staining confirmed a benign lipoma with no evidence of any malignant transformation. H&E = hematoxylin and eosin; CDK4 = cyclin-dependent kinase 4; MDM2 = mouse double minute 2 homolog.

**Figure 7:**
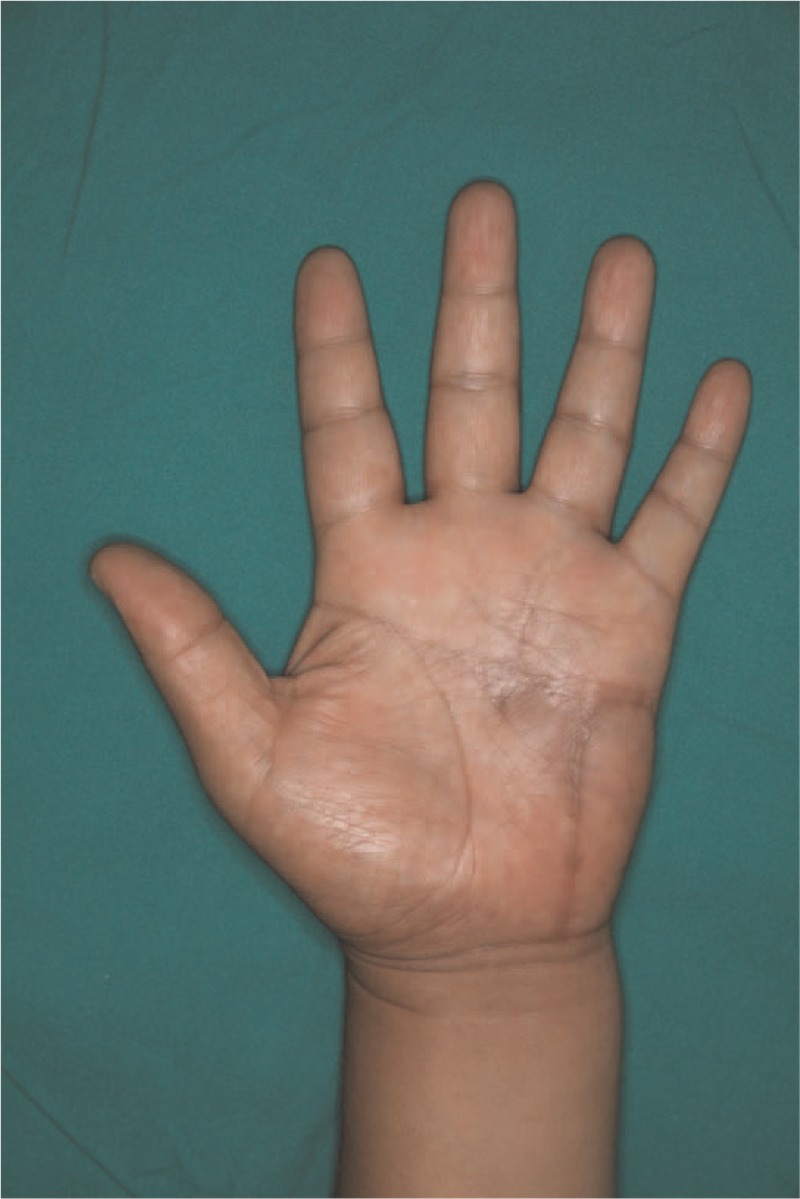
Photograph at 6 months after surgery. Complete sensory recovery was achieved 6 months after surgery, without any sign of recurrence.

With the exception of temporarily reduced sensation in the left ring and little fingers immediately after surgery, no particular complications were noticed. Basic histologic examination revealed mature white adipose tissue without atypia. Furthermore, fibrous septa were observed, while mitotic figures, necrosis, or lipoblasts were not detected. Immunohistochemical staining showed no cyclin-dependent kinase 4 (CDK4) or mouse double minute 2 homolog (MDM2) expression. These results ruled out the possibility of malignancy (Fig. 6). Complete sensory recovery was achieved 6 months after surgery, without any sign of recurrence (Fig. 7).

We obtained the patient's clinical and radiologic data. Informed written consent was obtained from the patient for publication of this case report and accompanying images. This study was approved by the Institutional Review Board of Chonnam National University Hospital (CNUH-2018-308) and was conducted in accordance with the principles of the Helsinki Declaration II.

## 3 Discussion

Lipomas are benign tumors composed of adipose tissue that can appear anywhere on the body. They can occur in the subcutaneous layer or at intramuscular or intermuscular sites.^[3]^ The affected locations can be the neck, trunk, shoulders and arms, and rarely, the hands or feet.^[4]^ A lipoma in the hand appears as a slow-growing, painless, and soft lump.

Ultrasonography (US), computed tomography (CT), and magnetic resonance imaging (MRI) are used to diagnose lipomas. The advantages of US are that it does not employ ionizing radiation, while producing fast results at a relatively low cost. CT and MRI are better at providing details about the mass and the surrounding anatomy of a lipoma than US.^[3]^ MRI, in particular, is an important tool because of its high diagnosis rate in finding masses in hands and wrists, at 94%. MRI results for benign lipomas show clear boundaries and follow the subcutaneous fat signal in all sequences, with very thin septations.^[5]^ In the patient described in this study, the MRI sequences followed the subcutaneous fat signal in all sequences and showed a septum, leading to the presumption of a benign lipoma. Furthermore, CT and MRI results showed that the lipoma partially extended into the carpal tunnel, the intermuscular site, and the intertendinous spaces. The goal of the operation, therefore, was to remove the mass with minimum damage to surrounding tissues.

Giant lipomas are > 5 cm in diameter and are extremely rare in the hand. If a mass effect is seen, surgical excision is necessary.^[4]^ Some of the signs of a mass effect are hand function disability and finger paresthesia.^[2]^ The case in this study showed a mass effect that caused tingling sensations on the tips of the ring and little fingers.

In addition, malignancy must be ruled out for giant lipomas. Liposarcomas are one of the most common soft tissue sarcomas, comprising 7% to 27% of all soft tissue sarcomas.^[6]^ Recent studies report that masses which are fast-growing, >5 cm, or located in the intramuscular area may be a risk factor for liposarcoma.[^6^,^7^] Although radiological images showed that this case of a giant lipoma had a low probability of malignancy, and actual histological test results showed it to be benign, complete excision was conducted for the reasons discussed above.

The majority of the cases reported in the literature have demonstrated that surgical excision of lipomas has good results for functional recovery. In this case, however, the palmar digital branches of the ulnar nerve for the ring and little fingers passed through the mass and the branch for the little finger was firmly attached to it. This may have been the cause of the tingling sensation in the ring and little fingers. Therefore, to enable a complete excision of the mass, the branch for the little finger was temporarily transected. After complete excision of the mass, the transected branch was coapted again under microscopy.

This is the first report of a giant lipoma enveloping the nerve in the hand that has been successfully excised by severing and reconnecting immediately the nerve. Although giant lipomas in the hand can extend to vital components such as neurovascular structures and tendons, meticulous en bloc resection can provide excellent results without any complications.

## Author contributions

**Conceptualization:** Kwang Seog Kim.

**Data curation:** Hyeok Lee, Dong Seob Lim.

**Formal analysis:** Jae Ha Hwang, Sam Yong Lee.

**Investigation:** Hyeok Lee, Dong Seob Lim.

**Methodology:** Jae Ha Hwang, Sam Yong Lee.

**Project administration:** Kwang Seog Kim.

**Writing – original draft:** Kwang Seog Kim, Hyeok Lee, Dong Seob Lim.

**Writing – review & editing:** Kwang Seog Kim.

Kwang Seog Kim orcid: 0000-0002-6766-4640.

Jae Ha Hwang orcid: 0000-0001-6992-8067.

Sam Yong Lee orcid: 0000-0002-3185-2519.
